# Analysis of the Functional Polymorphism in the Cytochrome P450 *CYP2C8* Gene rs11572080 with Regard to Colorectal Cancer Risk

**DOI:** 10.3389/fgene.2012.00278

**Published:** 2012-12-03

**Authors:** José M. Ladero, José A. G. Agúndez, Carmen Martínez, Gemma Amo, Pedro Ayuso, Elena García-Martín

**Affiliations:** ^1^Service of Gastroenterology, Hospital Clínico San Carlos, Instituto de Investigación Sanitaria del Hospital Clínico San CarlosMadrid, Spain; ^2^Department of Medicine, Medical School, Universidad ComplutenseMadrid, Spain; ^3^Department of Pharmacology, University of ExtremaduraCáceres, Spain; ^4^Department of Biochemistry and Molecular Biology, University of ExtremaduraCáceres, Spain; ^5^Fundación Instituto Mediterraneo Para el Avance en Biotecnología e Investigación SanitariaMálaga, Spain

**Keywords:** *CYP2C8*, colorectal cancer risk, polymorphisms, xenobiotic-metabolizing enzymes, biomarkers

## Abstract

In addition to the known effects on drug metabolism and response, functional polymorphisms of genes coding for xenobiotic-metabolizing enzymes (XME) play a role in cancer. Genes coding for XME act as low-penetrance genes and confer modest but consistent and significant risks for a variety of cancers related to the interaction of environmental and genetic factors. Consistent evidence supports a role for polymorphisms of the cytochrome P450 *CYP2C9* gene as a protecting factor for colorectal cancer susceptibility. It has been shown that CYP2C8 and CYP2C9 overlap in substrate specificity. Because CYP2C8 has the common functional polymorphisms rs11572080 and rs10509681 (CYP2C8*3), it could be speculated that part of the findings attributed to *CYP2C9* polymorphisms may actually be related to the presence of polymorphisms in the *CYP2C8* gene. Nevertheless, little attention has been paid to the role of the *CYP2C8* polymorphism in colorectal cancer. We analyzed the influence of the CYP2C8*3 allele in the risk of developing colorectal cancer in genomic DNA from 153 individuals suffering colorectal cancer and from 298 age- and gender-matched control subjects. Our findings do not support any effect of the CYP2C8*3 allele (OR for carriers of functional *CYP2C8* alleles = 0.50 (95% CI = 0.16–1.59; *p* = 0.233). The absence of a relative risk related to CYP2C8*3 did not vary depending on the tumor site. We conclude that the risk of developing colorectal cancer does not seem to be related to the commonest functional genetic variation in the *CYP2C8* gene.

## Introduction

In addition to the known effects on drug metabolism and response, functional polymorphisms of genes coding for xenobiotic-metabolizing enzymes (XME) play a modest but consistent role in cancer risk. Genes coding for XME act as low-penetrance genes and confer significant risks for a variety of cancers related to the interaction of environmental and genetic factors (Agundez, 2004). Cytochrome P450 (CYP) enzymes are involved in the metabolism of drugs and other xenobiotics. Two enzymes of the CYP2C subfamily, CYP2C8 and CYP2C9, have received special attention because they are involved in the metabolism of several commonly used drugs and are capable of activating carcinogens and mutagens. Consistent evidence supports a role for polymorphisms of the cytochrome P450 *CYP2C9* gene that produce decreased enzymatic activity as a protecting factor for colorectal cancer susceptibility (Martinez et al., 2001; Chan et al., 2004, 2009; Cleary et al., 2010; Northwood et al., 2010).

One of the mechanisms proposed for this protecting effect is based on the effect of non-steroidal anti-inflammatory drugs (NSAIDs). Inflammation is a known risk factor for colorectal cancer, and the use of NSAIDs constitute a potential means of decreasing inflammation in the colonic epithelium (Ulrich et al., 2006). For this reason, interindividual variability in the metabolism of NSAIDs may play a role in colorectal cancer risk (Bosetti et al., 2012). Two cytochrome P450 enzymes, namely CYP2C9 and, to a lesser extent, CYP2C8, play a major role in the metabolism of NSAIDs (for a review, see Agundez et al., 2009). CYP2C enzymes are expressed in human colon and in colorectal cancer cells (Martinez et al., 2002; Bergheim et al., 2005b; Garcia-Martin et al., 2006b). In addition CYP2C8 expression in colorectal cancer cells is inducible (Garcia-Martin et al., 2006b), and it has been postulated that altered expression of CYP2C enzymes might contribute to the development of colon cancer (Bergheim et al., 2005a).

Although it is known that CYP2C9 y CYP2C8 have common substrates including NSAIDs (Garcia-Martin et al., 2004; Martinez et al., 2005; Totah and Rettie, 2005), a putative association between *CYP2C8* polymorphisms and colorectal cancer risk has not been analyzed in detail. Only the effect of *CYP2C8* polymorphisms as modifying factors for the protective effect of regular NSAID use has been analyzed (McGreavey et al., 2005). This study indicated a lack of association of *CYP2C8* polymorphisms, but also of *CYP2C9* polymorphisms as modifiers of the protective effect of regular NSAID use on the risk of colorectal carcinoma. Nevertheless, the potential of *CYP2C8* polymorphisms as potential modifiers of colorectal cancer risk remains to be analyzed in detail.

In the present study we analyzed the association of CYP2C8*3 with colorectal cancer in a Spanish population. CYP2C8 is involved in the metabolism of arachidonic and retinoic acid. It is the main enzyme involved in the metabolism of R-ibuprofen and it has been shown to make a significant contribution to the metabolism of many other NSAIDs (Martinez et al., 2006). Several variant alleles have been described for the *CYP2C8* gene, CYP2C8*3 being the most common variant allele among Caucasian individuals (Garcia-Martin et al., 2006a). The effect *in vivo* of this mutation is a decrease in the metabolism and altered pharmacokinetics of CYP2C8 substrates (Martinez et al., 2005).

## Materials and Methods

The study group consisted of 153 unrelated consecutive patients (82 men and 71 women) with colorectal cancer, and 298 healthy subjects (Table 1). These subjects have participated in previous studies (Garcia-Martin et al., 2001; Martinez et al., 2001). All the participants were white Spanish individuals, living in the same areas as the patients (Madrid and surrounding areas), and were included in the study after giving informed written consent. The diagnosis was based on histology analyses of endoscopic biopsies and/or surgical resection specimens. Data regarding known previous digestive diseases, alcohol, and tobacco consumption, serum tests for hepatitis B and C virus and other diseases were collected. Heavy drinkers were defined as individuals drinking more than 50 g of alcohol per day. All the patients were requested to participate in the study, and all of them agreed to do so. Control samples were obtained from medical students, University, and Hospital staff. A medical examination was carried out to identify subjects in good health. Over 95% of the healthy subjects requested agreed to participate in the study. The protocol was approved by the Ethics Committee of the San Carlos University Hospital, Madrid. A possible confounding factor in the present study is that, within a study group, the frequency of individuals carrying a determined variant allele may change with age in the event that the presence of such an allele would be related to severe diseases. If a determined genotype has a “protecting effect” against any disease, it may be expected that in populations consisting of older subjects there is an increased frequency of such a protecting genotype. Therefore we included within the control group a selected subgroup of 41 healthy subjects with ages ranging from 90 to 95 years (Martinez et al., 2001). Genotype analysis indicates frequencies that were identical to those of younger healthy subjects (see Results). Another possible confounder is related to the fact that the control subjects are highly educated people, and differences in lifestyle as compared to cancer patients may be expected. Since digestive cancers are partly related to diet, these changes in lifestyle may be relevant. However, it should be stated that patients and controls were interviewed to assure that diet and lifestyle did not differ between patients and control subjects. Blood samples from all participants were stored at −80°C until analysis and genomic DNA was prepared from peripheral leukocytes. The analyses for the CYP2C8*3 gene variants were carried out by amplification-restriction procedures. Briefly, the pair of primers 5′-CTTCCGTGCTACATGATGACG-3′ and 5′-CTGCTGAGAAAGGCATGAAG-3′ was used for a mismatch PCR-RFLP test. The 117 bp PCR product was purified and digested with the endonuclease *Xmn*I, that produced two fragments of 92 and 25 bp in the CYP2C8*1 allele product, and did not digest the CYP2C8*3 PCR product. Further details are described elsewhere (Martinez et al., 2005). Twenty DNA samples with every genotype (CYP2C8*1/*1 and *1/*3) and all samples with the CYP2C8*3/*3 genotypes were cross-tested with TaqMan probes designed to detect the CYP2C8*3 variant allele (rs11572080; C__25625794_10; Applied Biosystems, Madrid, Spain) and in all cases the genotypes obtained by amplification-restriction and by the TaqMan analyses fully corresponded.

**Table 1:** **Characteristics of the individuals included in the study**.

	Patients		Controls	
	Male (*n* = 82)	Female (*n* = 71)	Male (*n* = 160)	Female (*n* = 138)
Age years (mean ± SD)	66.9 ± 9.6	65.6 ± 12.9	65.8 ± 10.2	66.0 ± 11.7
Non-smokers/smokers	53/29	69/2	108/52	129/9
Non-drinkers/drinkers	60/22	71/0	141/19	138/0
**TUMOR SITE**				
Non-sigmoid	33	22	–	–
Sigmoid	26	22	–	–
Rectum	23	27	–	–

### Statistical analysis

The intergroup comparison values were calculated using chi-square or Fisher’s exact tests when appropriate. Logistic regression analyses were performed using the SPSS 15.0 for Windows (SPSS Inc., Chicago, IL, USA) to test for confounders, including gender, age at diagnosis, smoking and drinking habits, and tumor localization. The 95% confidence intervals were also calculated. The statistical power calculated for the sample size of this study, determined from allele frequencies with a genetic model analyzing the frequency of the risk gene with RR = 2.0; *p* = 0.05) for two-tailed and one-tailed associations of the risk with the variant allele is 98.1 and 99.2%, respectively. The minimum odds ratio detectable with this sample size is 1.55, with a statistical power for one-tailed association equal to 80%.

## Results

Table 2 shows the CYP2C8*1/*3 genotypes and allele frequencies in colorectal cancer patients and healthy subjects. Differences noted by the comparison of genotype frequencies among them were not statistically significant. Odds ratio for carriers of the functional CYP2C8*1 allele = 0.50 95% C.I. = 0.160–1.59; χ^2^ = 1.42; *p* = 0.233. These findings were not influenced by gender, previous surgical therapy, or chemotherapy and Dukes’ stage of the tumor. Genotypes in both patients and control subjects are in Hardy–Weinberg’s equilibrium. Deviation test from Hardy–Weinberg’s equilibrium (Pearson): *p* = 0.172 (patients); *p* = 0.265 (controls). Trend test with the number of variant CYP2C8*3 alleles: *p* = 0.584.

**Table 2:** CYP2C8*1/*3 genotypes and allele frequencies in colorectal cancer patients and healthy controls.

	Patients (*n* = 153, 306 alleles)	Controls (*n* = 298, 596 alleles)	OR (95% CI), *p*
GENOTYPES rs11572080 C/T (*1/*3)			
CYP2C8*1/*1	111 (72.5; 65.5–79.6)	202 (67.8; 62.5–73.1)	1.26 (0.80–1.98), 0.299
CYP2C8*1/*3	36 (23.5; 16.8–30.3)	90 (30.2; 25.0–35.4)	0.71 (0.44–1.14), 0.135
CYP2C8*3/*3	6 (3.9; 0.8–7.0)	6 (2.0; 0.4–3.6)	1.99 (0.56–7.09), 0.233
**ALLELE FREQUENCIES**			
CYP2C8*1	258 (84.3; 80.2–88.4)	494 (82.9; 79.9–85.9)	1.11 (0.75–1.64), 0.586
CYP2C8*3	48 (15.7; 11.6–19.8)	102 (17.1; 14.1–20.1)	0.90 (0.61–1.33), 0.586

*The values in each cell represent: number (percentage) and [95% confidence intervals]*.

To elucidate whether the CYP2C8 genotype could be related to a particular type of colorectal cancer, patients were divided into three subgroups according to the anatomical site of the tumor (rectum, sigmoid colon, and non-sigmoid colon). Table 3 shows that none of the intergroup comparisons or comparisons with control subjects were significant. Deviation test from Hardy–Weinberg’s equilibrium (Pearson): *p* = 0.603 (non-sigmoid); *p* = 0.167 (sigmoid); *p* = 0.587 (rectum). Logistic regression analyses did not indicate any influence of confounders such as gender, age at diagnosis, smoking and drinking habits, or tumor localization in the negative findings obtained in this study.

**Table 3:** CYP2C8*1/*3 genotypes of patients with colorectal cancer according to tumor site.

	Non-sigmoid	Sigmoid	Rectum
GENOTYPES rs11572080 C/T (*1/*3)			
CYP2C8*1/*1	39 (70.9; 58.9–82.9)	37 (77.1; 65.2–89.0)	35 (70.0; 57.3–82.7)
CYP2C8*1/*3	14 (25.5; 13.9–37.0)	9 (18.8; 7.7–29.8)	13 (26.0; 13.8–38.2)
CYP2C8*3/*3	2 (3.6; 0–8.6)	2 (4.2; 0–9.8)	2 (4.0; 0–9.4)
**HAPLOTYPES**			
CYP2C8*1	92 (83.6; 76.7–90.5)	83 (86.5; 79.6–93.3)	83 (83.0; 75.6–90.4)
CYP2C8*3	18 (16.4; 9.5–23.3)	13 (13.5; 6.7–20.4)	17 (17.0; 9.6–24.4)

*The values in each cell represent: number (percentage) and (95% confidence intervals)*.

## Discussion

The clinical use of genetic biomarkers for the identification of high risk subpopulations is a major goal which scientists have long been pursuing. In the case of colorectal cancer, the search for risk biomarkers by means of genome wide association studies (GWAs) has provided relevant information, pointing in particular to the single nucleotide polymorphism rs3802842 at 11q23.1 and five additional loci. When SNPs in these loci are combined, the risk increases with an increasing number of variant alleles (Pittman et al., 2008; Tomlinson et al., 2008). Nevertheless, the biomarkers identified in GWA studies explain only a minor percentage of the risk and so these studies should be completed with additional research.

Various candidate gene association studies (CGAs) have been carried out in an attempt to identify low-penetrance genes capable of increasing the risk of developing colorectal cancer. With regard to cytochrome P450 enzymes, two mechanisms have been proposed to explain a putative association between the *CYP2C9* polymorphism and colorectal cancer risk. A number of xenobiotics that are ingested as pyrolysis products have been related to colorectal cancer risk. These include polycyclic aromatic hydrocarbons and heterocyclic aromatic amines. Several of these mutagenic substances are CYP2C substrates (Snyderwine et al., 1997; Jonsdottir et al., 2012) and therefore a genetically determined alteration in the metabolism of carcinogens and mutagens may underlie the observed association. This first hypothesis would imply that, besides CYPs, polymorphisms in other enzymes involved in the metabolism of polycyclic aromatic hydrocarbons and heterocyclic aromatic amines would influence the risk. This hypothesis is partly supported by the influence of *NAT2* polymorphisms in colorectal cancer risk (revised in Agundez, 2008). The second mechanism proposed to explain the association between the *CYP2C9* polymorphism and colorectal cancer risk is the key role that CYP2C9 plays in the metabolism of NSAIDs. This, together with the protective effect that NSAIDs play in human colorectal cancer, leads to the theory that in individuals with high CYP2C9 enzyme activity the protective effect of NSAIDs could be diminished. Because CYP2C8 and CYP2C9 show partial overlapping in substrate specificity, particularly related to some NSAIDs such as ibuprofen, celecoxib, diclofenac, or tenoxicam (Tang, 2003; Garcia-Martin et al., 2004; Martinez et al., 2005; Daly et al., 2007; Daily and Aquilante, 2009), it could be speculated that the presence of defect *CYP2C8* variant alleles may influence the risk of developing colorectal cancer.

To date, only the effect of polymorphisms in CYP2C8 as modifiers of the protective effect of regular NSAID use has been analyzed. (McGreavey et al., 2005) In this study, *CYP2C8* or *CYP2C9* polymorphisms did not influence the protective effect of regular NSAID use on the risk of colorectal cancer. Because *CYP2C8* and *CYP2C9* polymorphisms show a great interethnic and intraethnic variability (Martinez et al., 2006) and because positive association of *CYP2C9* genotypes with colorectal cancer risk was observed in some populations (Bigler et al., 2001; Martinez et al., 2001; Tranah et al., 2005; Hubner et al., 2006; Samowitz et al., 2006; Liao et al., 2007; Cotterchio et al., 2008; Cross et al., 2008; Chan et al., 2009; Siemes et al., 2009; Cleary et al., 2010) but not in other populations, and particularly in UK individuals (Sachse et al., 2002; Landi et al., 2005; Hubner et al., 2006; Kury et al., 2007). It could be speculated that the association of low-penetrance genes with colorectal cancer risk may vary from one population to another. Diet is a key factor in colorectal cancer risk, and because dietary determinants of colorectal cancer may vary greatly in different geographic locations, the contribution of low-penetrance genes to the overall risk may vary across populations.

For the above-mentioned reasons we decided to undertake the study in a population in which a positive effect of *CYP2C9* polymorphism in colorectal cancer risk had already been identified. Because CYP2C9*2 and CYP2C8*3 variant alleles are at linkage disequilibrium (Yasar et al., 2002; Martinez et al., 2005, 2007), it could be expected that the association observed between the CYP2C9 genotypes and colorectal cancer risk (Martinez et al., 2001; Chan et al., 2004, 2009; Cleary et al., 2010; Northwood et al., 2010), could be partly related to the presence of the CYP2C8*3 variant allele. However, the findings obtained in the present study indicate that no major association of the CYP2C8*3 variant allele and colorectal cancer risk is present in the population analyzed.

A limiting factor in this study is that no other defect *CYP2C8* alleles were analyzed. However, it should be emphasized that we tested for CYP2C8*3 which is the most common defect *CYP2C8* allele in Caucasian individuals (see the website http://www.cypalleles.ki.se/cyp2c8.htm), whereas other variant alleles such as CYP2C8*2, CYP2C8*4, or CYP2C8*5 are extremely rare or no conclusive evidence for their deleterious effect on drug metabolism *in vivo* is available, revised in (Martinez et al., 2006). Another putative limiting factor in this study is the sample size. It should however be emphasized that in the study population the positive association of colorectal cancer risk with *CYP2C9* genotype was identified with a smaller sample size (Martinez et al., 2001) and hence, in the event that a major linkage between CYP2C8*3 and colorectal cancer risk might exist in the population studied, the sample size would be enough to identify it. In conclusion, our findings, with sufficient statistical power to detect an odds ratio equal to 1.6, indicate that the CYP2C8*3 variant allele does not show a major association with colorectal cancer risk.

## Conflict of Interest Statement

The authors declare that the research was conducted in the absence of any commercial or financial relationships that could be construed as a potential conflict of interest.
